# Supplementary material for: Factors associated with early sexual debut among adolescents and youth in Mozambique: A geo-additive survival analysis of the Mozambique 2021 AIDS indicator survey
Source: PLoS One. 2025 Jun 25;20(6):e0326869. doi: 10.1371/journal.pone.0326869 (PMC12192294; doi:10.1371/journal.pone.0326869)
Supplement: Attachment — Submitted filename: Response to Reviewers.pdf [file pone.0326869.s001.pdf]

Factors associated with early sexual debut among adolescents and youth in Mozambique: a geo-additive survival analysis of the Mozambique 2021 AIDS indicator survey

Rachid Muleia, Shelsea Luís Damião, Áuria Ribeiro Banze, Cynthia Semá Baltazar and Isaac Akpor Adjei

We would like to express our appreciation for the thorough review of the manuscript from the reviewers. We have addressed all the comments and suggestions from the reviewers. It considerably improved the manuscript and helped us deepen our understanding about the topic. Below you can find point by point replies to the comments. The changes in the manuscript are highlighted in blue.

Reviewer 1

1. Given that a time to event approach is being used, some of the explanatory variables that are noted appeared to potentially be related to periods that occurred AFTER the event. For example, education, marriage, alcohol & drugs, living away from home etc. While the discussion section does acknowledge that the survey is cross-sectional rather than longitudinal, does the fact that some of these variables are only relevant outside of the hazard period raise a broader methodological question? The section talking about the link between marriage and early sexual debut for example got me thinking about what the relationship was between the sexual debut prior to 15 years and the fact that the marriage may be many years later and potentially unrelated to the sexual partner involved in the earlier event. Similar considerations are at play for employment - is this potentially many years after the early sexual debut rather than prior to the event? Perhaps this could be noted explicitly and/or made clear if these variables are actually all within the pre-event window.

It's true that these variable may be potentially related to the periods that occurred after the event. Additionally, from the survey data, it is not possible to tell weather they are within the pre-event window. Nevertheless, they serve as proxies for earlier life circumstances and behaviors, which can actually help us have some sense about the past conditions based on the current status.

For example, the current education level may reflect the individual's educational trajectory and access to education during their formative years, which, for instance, if the individual's current education is secondary, that may suggest that during the their adolescence they were engaged in school, and this may have influenced to delay their sexual debut. Similarly, the marital status can indicate social and cultural contexts that might have influenced early sexual debut. In many societies, the early marriage if often linked to early sexual debut. So, this can give us some idea on social environment of the participant during adolescence.

Regarding employment status, it can reflect socio-economic conditions and responsibilities that might have existed during adolescence. For example, if an individual is currently employed, it may suggest they had to assume adult roles and responsibilities early, potentially correlating with early sexual debut.

We recognize that some of these variables may not fall within the pre-event window, which is a limitation of the study, and the results should be interpreted with caution. However, this does not diminish their relevance in explaining early sexual debut, keeping in mind that they are proxies. This has been stated in the manuscript.

2. There are a couple of small typos or things to check - e.g. the formular for the exponential and gaussian covariance functions are the same, the phase "likewise Ganguli and Wand" may be better as "Similar to...", the label under figure 4 says "you" instead of "youth".

Thank you for this comment. We have corrected the errors.

3. In Ganguli & Wand, there was detail provided in relation to the number of knots used within the geospatial smoothing, however the current manuscript does not discuss this detail. The Discussion section however does mention that the 'geographical locations' are the enumeration level - is that what is being used for the knots, rather than a low-rank approach using only a subset of knots? (please excuse if I have misunderstood something here)

   Thank you for this observation. We used a low rank approach and with have clearly stated this in the manuscript.

4. In Ganguli & Wand, the contour plot of the geospatial factor focused on the log hazard ratio in order to draw attention to where geography seemed to impact the hazard ratio. Would this be worth considering in Figure 4? It's not quite clear what the contour scale is showing at the moment.

   Thank you for this observation. We have also focused on the log hazard ratio scale. We have mention this in the manuscript.

5. The socio-demographic profile notes that early debut is significantly more likely for males than females, but the Kaplan-Meier section immediately following calls out that this difference is not significant. Does that warrant a highlight?

   Indeed, we noted that early sexual debut is more common among males. It is important to emphasize that the variable "early sexual debut" is binary, and the test used is the Pearson Chi-square, which is sensitive to differences in proportions. These differences can occur due to study imbalance. In contrast, the Kaplan-Meier analysis examines the time to sexual debut, and the log-rank test used is sensitive to differences in the timing of the event. The non-significant result in the Kaplan-Meier analysis indicates that when considering the entire range of ages for sexual debut, the timing does not differ significantly between genders. Additionally, the sociodemographic profile was presented as exploratory to provide more context about the data.

6. Similar to 5, there are several references that appear to be conflicting in terms of whether northern/central/southern regions have higher vs lower hazard e.g. the prevalence rates in table 1 show the northern with a higher prevalence, similar with the Kaplan-Meier results, however the cox model results in table 3 suggest that the northern region (which is the reference) has a lower hazard rate than the others.

   Thank you for this observation, it helped us review our analysis. Actually, we noticed that it was redundant to include the variable region in the model as geographic differences can be accounted for by the spatial component in the model, which is modeled through radial basis splines. We have corrected this and all the results are aligned.

Reviewer 2

1. Abstract: Currently, the abstract talks in broad generalities, with no specifics. I appreciate why the authors might have done this, but I ask they consider at least a few numbers. What is the N of their study? What is the overall % of early sexual debut (or, if they wish, the regional % given the assertion of differences)? You don't need to provide too many details (I think it's fine, for example, to list correlates, without presenting your measures of effect), but at least tell us the size of your study, and your overall estimate(s). Abstract: I recommend that you specify the ages of your adolescent and youth data set (i.e., ages 15-24)

   Thank you for this suggestion. We have included this information in the Abstract

2. Methods, line 79: Why did you consider a subset and not the full data set? How did you select your subset? Your subset represents what percentage of the whole data set?

   Actually, we wanted to clarify that from the entire data set of individuals aged 15 and above, we only considered adolescents and youth aged 15 to 24 years, totaling 5,283 individuals. We apologize for any confusion, as the text may have implied that we considered a sub-sample from individuals aged 15 to 24 years.

3. Methods: can you make your R code and data set available as supplemental material? Your analysis is an interesting one, and I'm sure some readers may be intrigued to review your work and learn more.

   We have made the R code publicly available in a github repository at https://github.com/RachidMuleia/sexual_debut.git

4. Results: Minor point, but "had sex" should probably be "reported having had sex" or similar. Did you do anything to consider reporting bias? Younger participants may have been less confident or comfortable discussing sex and sexual debut, and may have been less likely to report sexual activity.

   Thank you. We have corrected it . With regard to reporting bais, considering this is a cross-sectional survey, it is expected that some participants might omit some information. Therefore, the results should interpreted bearing in mind that. We discuss it as a limitation of the study.

5. Results, line 220: Please be cautious with statements like "the prevalence is marginally higher" as the prevalence is statistically equivalent. I do not recommend making claims of modest or marginal difference, where this is really not the case (to me, a "modest" difference is a small, but statistically significant difference, where what you're showing is very clearly equivalent prevalence across both groups). Kindly go through your paper and consider removing any other claims of this nature.

   Thank you for this. We have gone through the text and corrected all such statement in the text.

6. Results, line 222: How do you define "high" vs "low" socio-economic status? I could not find this specified in your methods.

   Sorry for this. we have properly defined this in the method section.

7. Results line 242-3: I thought your outcome was early sexual debut, not just sexual debut? This sentence wasn't very clear to me. Your methods make clear that your

outcome is not sexual debut, yes/no, but rather age at sexual debut.

Indeed, the outcome is early sexual debut. We have corrected it every where in the text. Thank you for this observation.

8. Results, model selection. This section is very technical, and I'm not sure it brings much to the discussion of sexual debut. What is the importance/rational for comparing "circular, exponential, Gaussian, multiquadratic inverse, Matérn, and spherical covariance functions" and what is this telling us about age of sexual debut in Mozambique? Some of this section may be best suited to the discussion, or to a more detailed methods paper to describe why you selected the model you did. I don't think this belongs here in the results, however – or perhaps in a less technical, distilled sentence or two. (Same with Table 2)

   Thank you for this observation. We have removed this section and described the motivation for choosing spherical semivariogram in the method section.

9. Results, "Covariate effects": Are you modeling "sexual debut" as a bivariate, yes/no, outcome (e.g., lines 281, 283, 284, etc)? That's how this section reads. However, your methods section makes it sound like you are modeling time to sexual debut? Or early sexual debut (i.e., sex before age 15), yes/no? Please clarify.

   We are modellig time to sexual debut and we have made it clear in the text.

10. Results, spatial trend: I would encourage the authors to avoid technical jargon in the results. It's good to explain what you did in the methods, but in the results section, I recommend stating what you found (only). I can refer back to the methods if I wish for more details. (i.e., edit to remove "visualization of the radial basis spline" and "overall geoadditive model" etc)

    Thank you for this observation. We have removed the technical jargon in the result section.

11. Discussion: The authors seem torn between presenting their results, and discussing the

merits of their modeling approaches. The whole first paragraph of your results re-states your modeling strategy! If I'm hear to learn about early sexual debut in Mozambique, I likely have little interest (or background to understand) why you adopted one strategy over another. I think you may have two papers, here, a methods paper (where you can go much deeper) and a results paper (where you really should limit much of your discussion of methods). Start your discussion with a high level summary of what you found (ie., remove the first paragraph of the discussion –I am guessing most with an interest in the topic would want to start with your second paragraph).

Thank you for this observation. We have revomed the first paragraph of the discussion, focusing more on reporting the summary findings.

Editor

1. Please ensure that your manuscript meets PLOS ONE's style requirements, including those for file naming.

   We have tried follow all of the journal's requirements.

2. 2. In the online submission form, you indicated that the survey data set is publicly available at https://phia.icap.columbia.edu/surveys/ upon reasonable request. For the current study, we considered a sub-sample of 5283 adolescents and youth aged 15-24 who consented to participate.

   We have provided the data and the code used in the manuscript and the data can be found at (`https://github.com/RachidMuleia/sexual_debut.git`)

3. We note that Figure 4 in your submission contain [map/satellite] images which may be copyrighted. All PLOS content is published under the Creative Commons Attribution License (CC BY 4.0), which means that the manuscript, images, and Supporting Information files will be freely available online, and any third party is permitted to access, download, copy, distribute, and use these materials in any way, even commercially, with

proper attribution. For these reasons, we cannot publish previously copyrighted maps or satellite images created using proprietary data, such as Google software (Google Maps, Street View, and Earth). For more information, see our copyright guidelines: http://journals.plos.org/plosone/s/licenses-and-copyright.

We require you to either (1) present written permission from the copyright holder to publish these figures specifically under the CC BY 4.0 license, or (2) remove the figures from your submission:

With regard to Figure 4, the shapefile used to produce this figure is available at `https://data.humdata.org/dataset/cod-ab-moz`. And according to their police all the data available at their website is licensed under a Creative Commons Attribution 4.0, which means that can be freely downloaded and distributed.

4. Please include a copy of Table 2 and 4 which you refer to in your text on page 9.

   We have provided table 2. With regard to Table 4 this was a mistake we don't have Table 4 in the manuscript. We have corrected this
