# Supplementary material for: Factors associated with early sexual debut among adolescents and youth in Mozambique: A geo-additive survival analysis of the Mozambique 2021 AIDS indicator survey
Source: PLoS One. 2025 Jun 25;20(6):e0326869. doi: 10.1371/journal.pone.0326869 (PMC12192294; doi:10.1371/journal.pone.0326869)
Supplement: Attachment — Submitted filename: Response_to_Reviewers.pdf [file pone.0326869.s002.pdf]

Factors associated with early sexual debut among adolescents and youth in Mozambique: a geo-additive survival analysis of the Mozambique 2021 AIDS indicator survey

Rachid Muleia, Shelsea Luís Damião, Áuria Ribeiro Banze, Cynthia Semá Baltazar and Isaac Akpor Adjei

We would like to once again express our sincere appreciation to the reviewers and the editor for their thorough and constructive evaluation of our manuscript. We have carefully addressed all the comments and suggestions provided, which we believe have significantly improved the quality of the manuscript and deepened our understanding of the topic. Below, we provide detailed, point-by-point responses to each comment. All changes made to the manuscript are highlighted in blue

Reviewer 1

1. I observed a small number of typographical errors that would be worth fixing if possible - e.g. the figure of 18.9% early debut sex is also quoted as 18.8% elsewhere in the paper. There is also a typo ”individual male individual” and the word ”analyzes”. Small observations.

   Thank you for this observation, we have corrected it. .

Editor

1. Clearer: The variables measure current conditions at the time of the survey so that they reflect events happening after the sexual debut.

   Thank you for this observation, we have clearly stated this in the manuscript

2. 2. More comprehensive: You have mentioned some of the least problematic variables on that respect. I think there are three variables that are more problematic: - Sex of household head: I would drop it from the analysis. I do not know what is the rationalle, but note how this would be different in married/divorced from unmarried adolescents living with their parents. It will confound different effects. You are not providing any rationale. I would remove it overall from the study.

   Thank you for this observation, we have removed the variable Sex of the household from the analysis

3. Recent consumption of alcohol/illicit drugs. In this case there could be reversed causality.

   Thank you for this observation, this was not clear before as we only thought that the relationship could be in one direction, but now we have a deeper understanding of reverse causality concept and we have properly mentioned this in the manuscript as it's implication in the analysis.

4. Marital status: You have mentioned this as a factor for early sexual debut and, in this case, you expect a causal effect from early marriage to early sexual debut. It is true that marriage behaviour could also adapt after early sexual debut leading to reverse causality.

   Tank you for this observation, we have addressed this in the manuscritp

5. As commented: make clear, and more comprehensive. Include the words reverse causality where appropriate.

   Thank you for this observation. We have properly included reverse causality where needed.

We have also taken this opportunity to check the reference list and we noted some reference were missing.
